# Editorial: Applications of fluorescence in surgery and diagnostics, volume II: evolution and breakthroughs

**DOI:** 10.3389/fsurg.2026.1806002

**Published:** 2026-03-11

**Authors:** Evgenii Belykh, Mark C. Preul

**Affiliations:** 1 Department of Neurosurgery, New Jersey Medical School, Rutgers University, Newark, NJ, United States; 2 The Loyal and Edith Davis Neurosurgical Research Laboratory, Department of Neurosurgery, Barrow Neurological Institute, St. Joseph's Hospital and Medical Center, Phoenix, AZ, United States

**Keywords:** applications, breakthroughs, diagnostics, fluorescence, surgery, confocal laser endomicroscopy

## Introduction

Augmentation of surgical and diagnostic vision through fluorescence and advanced optical imaging is redefining intraoperative decision-making across surgical subspecialties. Fluorescence-guided techniques enable real-time visualization of biological processes, revealing tissue characteristics imperceptible to the unaided human eye. These approaches promise to improve the precision, safety, and efficiency of surgical interventions. In *Applications of Fluorescence in Surgery and Diagnostics Volume II: Evolution and Breakthroughs*, we present a focused collection of 11 open-access articles that reflect the evolution of fluorescence technologies and highlight key breakthroughs in fluorophores, imaging platforms, data interpretation, and clinical workflows.

**Figure 1:**
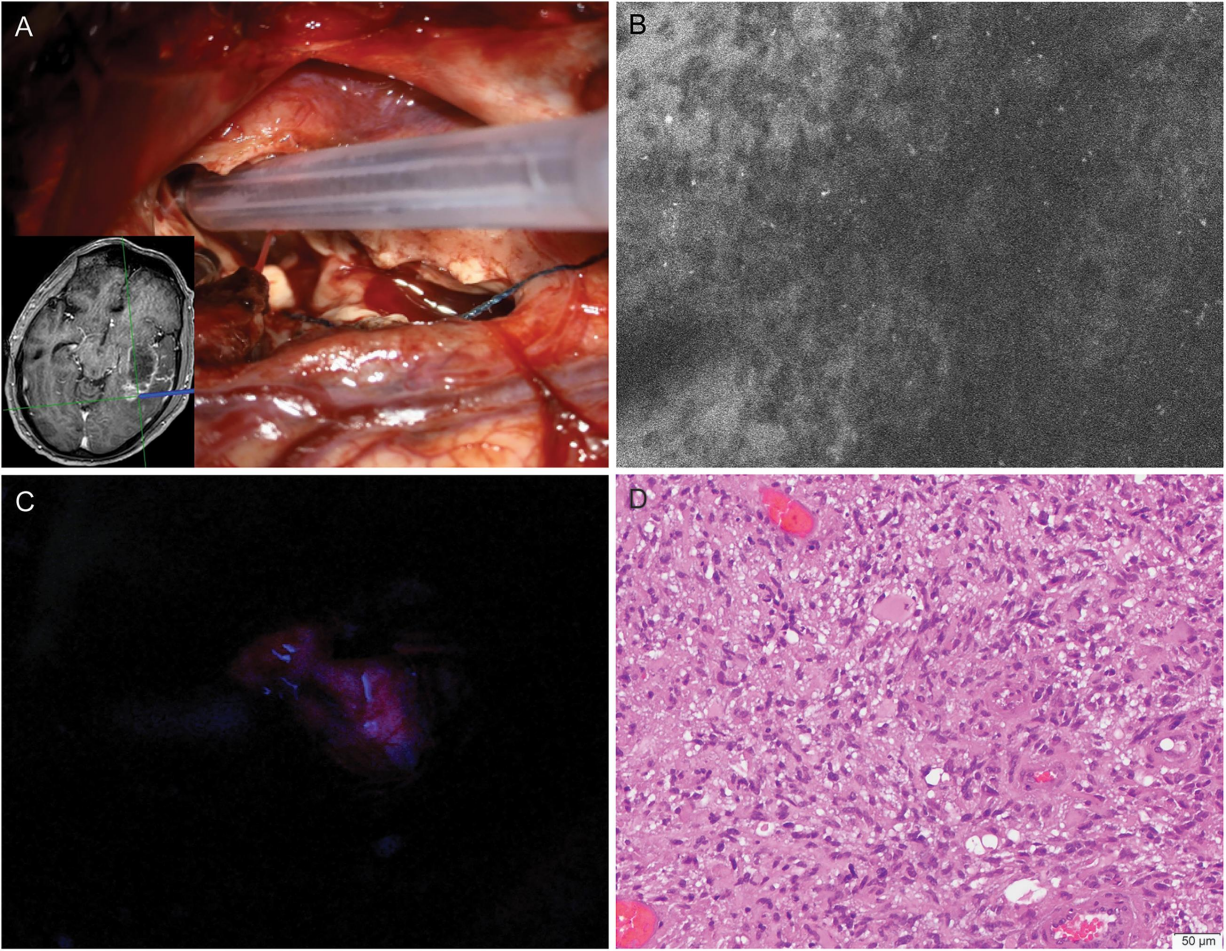
State-of-the-art fluorescence imaging technologies are used during the intraoperative management of a high-grade glioma. Confocal laser endomicroscopy (CLE) with cellular resolution and 5-aminolevulinic acid (5-ALA) wide-field operating microscope imaging are used to scan the deep margin during a glioma resection to assess for residual tumor that may require resection. **(A)** Intraoperative photograph showing the CLE probe tip at the deep tumor margin. Navigation MRI (*inset*) shows the extent of tumor and the location of CLE imaging at the probe tip. **(B)** Fluorescein-based CLE image reveals the typical appearance of a high-grade glioma with hypercellularity and pleomorphism. **(C)** Operating microscope view using Zeiss BLUE 400 imaging mode of the same deep marginal region shows 5-ALA-induced protoporphyrin IX grade 1 fluorescence. **(D)** Hematoxylin-and-eosin-stained biopsy specimen taken from the deep tumor margin, colocalized with the CLE image, reveals hypercellularity with numerous atypical cells, demonstrating concordance between fluorescence imaging techniques and histology. The CLE image correlates more closely with the characteristics of the biopsied tumor tissue evident than the protoporphyrin IX grade 1 fluorescence suggests. *Used with permission from Barrow Neurological Institute, Phoenix, Arizona*.

These contributions span preclinical investigation, translational research, clinical studies, systematic reviews, and case reports. They emphasize the importance of established fluorophores, such as 5-aminolevulinic acid, fluorescein sodium, and indocyanine green (ICG) and the rapid emergence of cellular-resolution imaging, artificial intelligence–assisted interpretation, and novel molecular probes that address unmet clinical needs (Figure 1). Neurosurgical technology use is rapidly expanding to optimize surgical resection of invasive brain tumors.

Xi et al. present real-world clinical evidence supporting fluorescein-guided surgery for resecting high-grade gliomas in “*Fluorescein-guided surgery for high-grade glioma resection: a five-year-long retrospective study at our institute*”. They found that fluorescein guidance was associated with higher gross total resection rates and lower postoperative residual tumor volumes, without increasing operative time, blood loss, or complication rates. For tumors in noneloquent regions, such as the temporal and occipital lobes, fluorescein sodium was a practical and accessible adjunct to standard microsurgical workflows.

Xu et al. address the critical question of who can reliably interpret intraoperative confocal laser endomicroscopy (CLE) images in “*Intraoperative in vivo confocal endomicroscopy of the glioma margin: performance assessment of image interpretation by neurosurgeon users*”. They show that neurosurgeons performed comparably to neuropathologists when interpreting CLE images at glioma margins, supporting CLE as a practical intraoperative guidance tool. Validating a simple dichotomous scoring system further enhances its feasibility for real-time use during surgery.

Brielmaier et al. investigate the biological basis of fluorescein staining patterns observed during brain tumor CLE in “*Fluorescein-distribution in confocal laser endomicroscopy allows for discrimination between primary brain tumours and metastases*”. Their combined *in vitro*, *ex vivo*, and *in vivo* analyses reveal tumor entity–specific intracellular fluorescein accumulation, enabling discrimination between primary brain tumors and metastatic lesions. This work highlights how fluorophore–tissue interactions enhance diagnostic interpretation beyond mere signal presence.

Artificial intelligence (AI) is rapidly progressing in fluorescence imaging assessment. Chen et al. introduce a sequence-based AI model that incorporates temporal information from CLE image streams, mimicking expert human interpretation in “*Artificial intelligence prediction of nonenhancing brain tumor malignancy based on in vivo confocal laser endomicroscopic imaging*”. Their approach outperforms frame-based models and achieves diagnostic accuracy comparable to that of neuropathologists. This study demonstrates that AI can reduce subjectivity, enhance specificity, and support intraoperative decision-making in the management of these tumors.

Xu et al. examine and assess the growing body of clinical evidence supporting CLE use in neurosurgery in “*Clinical application of confocal laser endomicroscopy in neurosurgery: a scoping review*”. CLE demonstrates diagnostic performance comparable to frozen-section pathology across multiple platforms and fluorophores, with faster feedback and seamless workflow integration. The authors emphasize the need for prospective interventional trials to define CLE's impact on surgical outcomes.

Zhang et al. report the extension of fluorescence guidance beyond neuro-oncology in “*A preliminary investigation of precise visualization, localization, and resection of pelvic lymph nodes in bladder cancer by using indocyanine green fluorescence-guided approach through intracutaneous dye injection into the lower limbs and perineum*”. They conceptualize a novel intracutaneous ICG injection strategy for pelvic lymph node mapping in bladder cancer. Their findings show high accuracy in lymph node identification and higher nodal yield than conventional approaches. This work illustrates how innovative delivery routes and imaging integration expand the diagnostic and therapeutic values of established fluorophores in oncologic surgery.

Schiena et al. solve a technical challenge using ICG fluorescence-enabled identification and ligation of the thoracic duct via contralateral video-assisted thoracoscopic surgery in “*Case report: thoracic duct ligation for left-sided chylothorax after pneumonectomy with contralateral VATS procedure using indocyanine green fluorescence*”. They underscore the value of fluorescence and demonstrate that ICG can facilitate minimally invasive solutions when conventional visualization is inadequate.

Several papers investigate advances in fluorescence-based technologies in preclinical laboratory settings. Belykh et al. present a systematic comparative analysis of three fluorophores in glioma models of various malignancy grades, using wide-field operative microscopy and confocal microscopy in “*5-Aminolevulinic acid, fluorescein sodium, and indocyanine green for glioma margin detection: analysis of operating wide-field and confocal microscopy in glioma models of various grades*”. Their work highlights the limitations of wide-field fluorescence in low-grade gliomas and that confocal imaging substantially improves sensitivity and diagnostic accuracy, particularly for detecting low concentrations of protoporphyrin IX and fluorescein sodium. This study reinforces the concept that no single fluorophore or imaging modality can delineate all tumor boundaries, underscoring the need for multimodal, multiscale imaging strategies.

Lin et al. present a next-generation near-infrared II viscosity-responsive probe designed to improve visualization of high-grade glioma tumor margins in “*A novel near-infrared II viscosity-responsive probe for surgical fluorescence guidance: laboratory investigation in a murine subcutaneous glioma model*”. Their preclinical data demonstrate superior photostability and tissue responsiveness than ICG, underscoring the potential of physicochemical tumor sensing. The study identifies future directions for probe design and translational imaging.

Shimizu et al. review the rapidly evolving landscape of fluorescent aminopeptidase probes, including HMRG- and 2MeSiR-based agents, with a focus on neurosurgical applications in “*Advancement of fluorescent aminopeptidase probes for rapid cancer detection–current uses and neurosurgical applications*”. They detail how these probes provide enzyme-activated, tumor-specific fluorescence and rapid signal generation as an alternative to metabolism-based contrast agents such as 5-ALA. This work highlights the shift in molecular probe design to higher specificity and cellular-level functional imaging.

Xu et al. demonstrate a novel application of fluorescein sodium as a marker of focused ultrasound–induced blood–brain barrier disruption in “*Fluorescein sodium as a marker for focused ultrasound-induced blood–brain barrier disruption: a case report in a porcine model*”. They correlate macroscopic and microscopic fluorescence findings with contrast-enhanced MRI and histology to assess real-time blood-brain-barrier permeability, with implications for drug delivery and intraoperative diagnostics.

Collectively, the articles in this volume illustrate how fluorescence-guided surgery and diagnostics are advancing synergistic developments in molecular probes, imaging platforms, interpretive frameworks, and computational tools. As these technologies mature, interdisciplinary collaboration will be essential to translate innovation into improved patient outcomes. Large-scale patient trials are necessary to demonstrate the practical utility of these techniques and to influence improved surgical outcomes and patient survival. This collection contributes to that effort, capturing the current state of the field and pointing to its future.

